# GPCR-SSFE: A comprehensive database of G-protein-coupled receptor template predictions and homology models

**DOI:** 10.1186/1471-2105-12-185

**Published:** 2011-05-23

**Authors:** Catherine L Worth, Annika Kreuchwig, Gunnar Kleinau, Gerd Krause

**Affiliations:** 1 Leibniz-Institut für Molekulare Pharmakologie (FMP), 13125 Berlin, Germany; 2 Structural Bioinformatics Group, Charité - Universitätsmedizin, 13125 Berlin, Germany; 3 Institut für Experimentelle Pädiatrische Endokrinologie, Charité - Universitätsmedizin, Augustenburgerplatz 1, 13353 Berlin, Germany

## Abstract

**Background:**

G protein-coupled receptors (GPCRs) transduce a wide variety of extracellular signals to within the cell and therefore have a key role in regulating cell activity and physiological function. GPCR malfunction is responsible for a wide range of diseases including cancer, diabetes and hyperthyroidism and a large proportion of drugs on the market target these receptors. The three dimensional structure of GPCRs is important for elucidating the molecular mechanisms underlying these diseases and for performing structure-based drug design. Although structural data are restricted to only a handful of GPCRs, homology models can be used as a proxy for those receptors not having crystal structures. However, many researchers working on GPCRs are not experienced homology modellers and are therefore unable to benefit from the information that can be gleaned from such three-dimensional models. Here, we present a comprehensive database called the GPCR-SSFE, which provides initial homology models of the transmembrane helices for a large variety of family A GPCRs.

**Description:**

Extending on our previous theoretical work, we have developed an automated pipeline for GPCR homology modelling and applied it to a large set of family A GPCR sequences. Our pipeline is a fragment-based approach that exploits available family A crystal structures. The GPCR-SSFE database stores the template predictions, sequence alignments, identified sequence and structure motifs and homology models for 5025 family A GPCRs. Users are able to browse the GPCR dataset according to their pharmacological classification or search for results using a UniProt entry name. It is also possible for a user to submit a GPCR sequence that is not contained in the database for analysis and homology model building. The models can be viewed using a Jmol applet and are also available for download along with the alignments.

**Conclusions:**

The data provided by GPCR-SSFE are useful for investigating general and detailed sequence-structure-function relationships of GPCRs, performing structure-based drug design and for better understanding the molecular mechanisms underlying disease-associated mutations in GPCRs. The effectiveness of our multiple template and fragment approach is demonstrated by the accuracy of our predicted homology models compared to recently published crystal structures.

## Background

Due to their fundamental role in signal transduction, G protein-coupled receptors (GPCRs) are the target of a large proportion of medical drugs. GPCR structural data are important for understanding the molecular mechanisms underlying diseases caused by mutations in these receptors, as well as for structure-based drug design. Up until November 2010, experimentally determined three-dimensional structures of family A GPCRs were only available for four members - see Topiol and Sabio [1] for a review of these structures. This is in stark contrast to the total number of family A GPCRs, which for humans alone has recently been estimated at 750 receptors [2]. All of the family A GPCR structures share a common molecular architecture of seven transmembrane helices (TMHs), therefore the deficit in GPCR experimental structures can be rectified by building molecular models of GPCRs of unknown structure using homology modelling techniques [3]. Currently, there are seven different GPCR structures to choose from when building homology models of GPCRs in the inactive state: bovine rhodopsin (Swiss-Prot:opsd_bovin) [4]; Japanese flying squid rhodopsin (Swiss-Prot:opsd_todpa) [5,6]; common turkey beta-1 adrenergic receptor (Swiss-Prot:adrb1_melga) [7]; human beta-2 adrenergic receptor (Swiss-Prot:adrb2_human) [8,9]; human adenosine receptor A2A (Swiss-Prot:aa2ar_human) [10]; human dopamine D3 receptor (Swiss-Prot:drd3_human) [11] and human CXCR4 chemokine receptor (Swiss-Prot:cxcr4_human) [12]. However, despite the common general architecture of these structures, key differences do exist between them. The choice of which experimental GPCR structure(s) to use for building a comparative model of a particular GPCR is unclear and without detailed structural and sequence analyses, could be arbitrary.

In order to help clarify the choice of template selection for GPCR homology modelling, we recently developed a workflow for identifying the most appropriate template to use for building homology models of family A GPCRs [13]. We analyzed in detail conserved and unique sequence motifs and structural features in the five experimentally-determined GPCR structures available at the time. Our analysis provided deeper insight into specific and important structural features of GPCRs and valuable information for template selection. Using key features, we formulated a workflow for identifying the most appropriate template(s) for building homology models of GPCRs of unknown structure. Our analysis revealed that the available crystal structures represent only a subset of all possible structural variation in family A GPCRs. Some GPCRs have structural features that are distributed over different crystal structures or which are not present in the templates, suggesting that homology models should be built using a fragment approach whereby template selection is carried out for each TMH, rather than the receptor as a whole.

There are a number of web resources available which focus on GPCRs. The IUPHAR database of GPCRs provides a comprehensive catalogue of peer-reviewed pharmacological, chemical, genetic, functional and anatomical information on human, rat and mouse nonsensory GPCRs, as well as links to other relevant resources [14]. The GPCRDB is an online information system for GPCRs which provides multiple sequence alignments, mutation data, ligand binding data and various tools such as an alignment builder and predicting the effects of mutations [15]. Interactions between human GPCRs, G proteins and effectors can be visualized using human-gpDB [16] whilst GPCR oligomer information is provided by GPCR-OKB [17]. Where structural data are provided by the aforementioned resources, they are limited to the handful of GPCRs with crystal structures available. The specialized GPCR databases SSFA [18] and GRIS [19] provide homology models of GPCRs, although these are restricted to the three glycoprotein hormone receptors. Zhang and co-workers used their threading assembler refinement method to build molecular models of 907 human GPCRs [20]. Although the models are available for download, they are currently not stored in a database and they were generated at a time when only the structure of bovine rhodopsin was available.

We have now extended our previous theoretical work and automated our workflow and applied it to a set of 5025 family A GPCR sequences. Based on these automated template suggestions, homology models were built of the seven TMHs and helix 8 of the 5025 receptors. Here, we present the GPCR-Sequence Structure Feature Extractor (SSFE) database, which stores the template predictions, the identified sequence and structure motifs and homology models of 5025 family A GPCRs. Users are able to browse the GPCR dataset stored in GPCR-SSFE or search for results using a UniProt entry name [21]. It is also possible for a user to submit a GPCR sequence that is not contained in the database for analysis, with the template suggestions for the seven TMHs and helix 8 returned along with a molecular model. The molecular models presented in GPCR-SSFE represent the most comprehensive set of GPCR models created so far and offer a valuable starting point for *in silico *functional predictions, docking studies and structure-based drug design.

## Construction and content

### GPCR dataset

GPCR-SSFE contains all family A GPCRs sequences that were stored in the GPCRDB as of October 2009, totalling 5025 receptors [15].

### Template selection and homology modelling

A profile hidden markov model (HMM) was used to align the 5025 GPCRs to the five template structures available at the time (before November 2010) (PDB codes: 1U19, 2Z73, 2VT4, 2RH1 and 3EML) [22]. HMMER2 http://hmmer.janelia.org/ was used to generate the profile HMM from a multiple sequence alignment (MSA) of the five templates plus 54 other family A GPCRs (see additional file 1 for a list of the GPCRs used to produce the HMM). The 54 GPCRs were selected so as to maximize the coverage of the phylogenetic tree for family A GPCRs [23]. After alignment, template selection was carried out for each TMH and helix 8 based on our previously published workflow [13], which for the purposes of this work was automated using python scripts. The workflow uses the presence or absence of structural features such as proline distortions, disulphide bridges and insertions as well as sequence similarity comparisons to select the best template(s) for modelling a given GPCR. Modeller 9v7 was used to generate homology models of query GPCRs using the alignments and templates suggested by GPCR-SSFE [24]. Three models were produced for each GPCR but only the one with the lowest DOPE score [25] is stored in GPCR-SSFE.

### Database implementation

GPCR-SSFE was built by combining an Apache web server http://www.apache.org, PHP Hypertext Pre-processor scripts (PHP 5) and a relational database management system (MySQL 5). The web interface is based on HTML, JavaScript and Cascading Style Sheets (CSS). The GPCR homology model structures are displayed using the Jmol structure viewer (Jmol: an open-source Java viewer for chemical structures in 3D http://www.jmol.org/.

## Utility

### The GPCR-SSFE website

**Figure 1:**
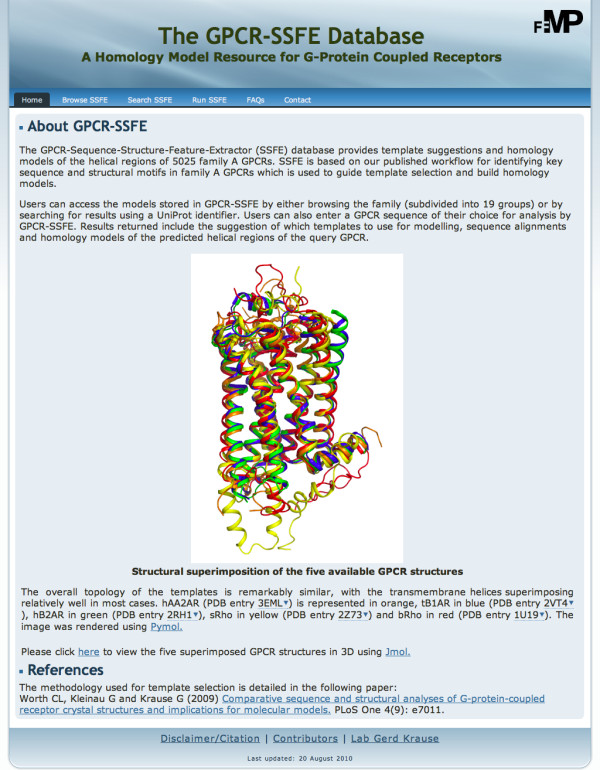
**The home page of the GPCR-SSFE database**. The menu at the top of the page allows users to easily navigate the site. The background to the database is provided, along with information about the templates used for homology modelling.

**Figure 2:**
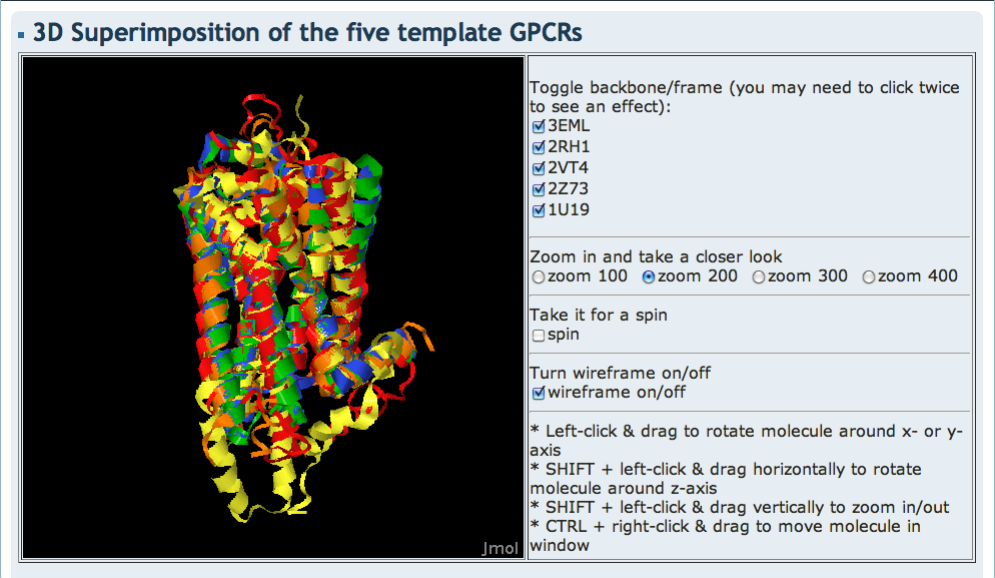
**Structural superimposition of the GPCR template structures**. Users can access a Jmol applet displaying the five crystal structures used for analysis and homology modelling via the home page.

The GPCR-SSFE has a user-friendly interface with a menu and a facts and questions (FAQ) page to aid usability. On the homepage of GPCR-SSFE (Figure 1), users can find information about the database and the template structures used for analysis and homology modelling, including a link to a Jmol applet showing the five template structures superimposed onto one another (Figure 2).

### Searching GPCR-SSFE

**Figure 3:**
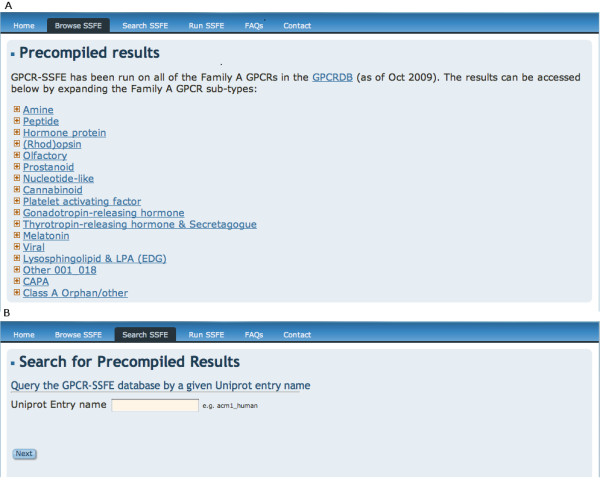
**Querying the GPCR-SSFE database**. Results may be retrieved from the database by either A) browsing the database via a list of 19 family A GPCR subgroups or B) by searching using a UniProt entry name.

Results can be retrieved by either browsing or searching the database. By navigating to the "Browse SSFE" menu option, the user is presented with a list of 19 family A GPCR subgroups (Figure 3A): Amine, Peptide, Hormone protein, (Rhod)opsin, Olfactory, Prostanoid, Nucleotide-like, Cannabinoid, Platelet activating factor, Gonadotropin-releasing hormone, Thyrotropin-releasing hormone & Secretagogue, Melatonin, Viral, Lysophingolipid & LPA (EDG), Leukotriene B4 receptors, Ecdysis triggering hormone receptor, Other, CAPA and Class A orphan/other. These subgroups correspond to those used in the GPCRDB and are based on the pharmacological classification of GPCRs. Each subgroup can be expanded by clicking on it, revealing either further subgroupings or (if the final node is reached) a list of GPCRs within the subgroup. Clicking on the UniProt entry name of a GPCR will take the user to the results page for that particular receptor. Alternatively, users may retrieve results by entering the UniProt entry name onto the "Search SSFE" webpage (Figure 3B).

### GPCR-SSFE results page

**Figure 4:**
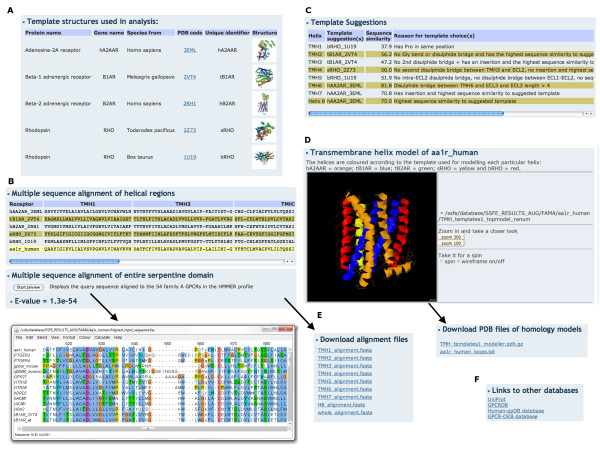
**GPCR-SSFE template prediction and homology model results**. Results are shown for adenosine receptor A1 and include A) the templates used for analysis and links to the PDB; B) the multiple sequence alignments of the individual helices and the alignment spanning the entire serpentine domain; C) The template predictions, sequence similarity scores and prediction reasons; D) A Jmol applet displaying the homology model of the query GPCR; E) Downloadable files of the alignments, loops and homology model and F) links to entries in other relevant databases.

The results returned by GPCR-SSFE include (Figure 4): i) the templates used for analysis and links to the PDB [26]; ii) the multiple sequence alignment (MSA) of the five templates and the query sequence for each individual transmembrane helix and helix eight; iii) the MSA of the profile HMM GPCR sequences and the query sequence, which spans the entire serpentine domain; iv) the HMMER2 e-value assigned to the full-length MSA; v) the template suggestions (and reasons) for the seven TMHs and helix eight; vi) the sequence similarity score between the suggested template(s) and the query sequence; vii) the rationale for the suggested templates; viii) a Jmol applet displaying the homology model(s) of the query GPCR based on the template suggestions; ix) the amino acid sequences of the intracellular and extracellular loops and x) links to UniProt, the GPCRDB and, where applicable, to the Human-gpDB and GPCR-OKB databases. The loops are not modelled and therefore the loop sequences are provided to the user so that software such as SuperLooper may be used for modelling these regions [27]. Files containing results ii, iii, viii and ix are made available for download (Figure 4E). Additionally, for stereochemical quality checking a PROCHECK [28] generated Ramachandran plot of each model is provided and information about how to interpret the results are provided on the FAQ page.

### Running GPCR-SSFE

**Figure 5:**
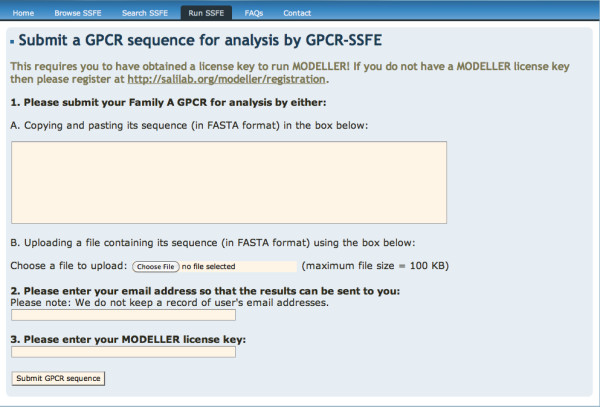
**GPCR-SSFE sequence submission page**. Where a family A GPCR is not contained in the database, a user may submit their sequence of interest to GPCR-SSFE for template prediction and homology modelling. Users must obtain a licence key for Modeller before they are able to use this function.

In some instances, GPCR-SSFE might not store results for particular family A GPCRs e.g. newly identified orphan GPCRs. For such cases, users can submit their GPCR sequence (in FASTA format) to GPCR-SSFE for analysis and homology model building by navigating to the "Run SSFE" webpage (Figure 5). Modeller 9v7 is used for homology modelling, therefore, users must obtain a Modeller license key before they can use this tool. This key is freely available for academic users and easily obtainable at: http://salilab.org/modeller/registration.shtml. To start the template analysis and homology modelling, the user enters their GPCR sequence onto the webpage (by either uploading or copying and pasting it), along with their Modeller licence key and email address. Once the results are ready, a web-link is emailed to the user. Results are stored on the server for seven days. The results page looks exactly like those retrieved when searching the database, except that links to external databases are not provided.

### Performance: A case study

Since completion of the large-scale GPCR model building and submission of the manuscript, two new family A GPCR crystal structures were published - the human dopamine D3 receptor (Swiss-Prot:drd3_human) [11] and the CXCR4 chemokine receptor (Swiss-Prot:cxcr4_human) [12]. These receptor structures therefore provide us with an ideal means of assessing the performance of the models produced by GPCR-SSFE as they were not included in the pool of crystal structure templates used by GPCR-SSFE and we can now calculate the RMSD of our predicted models to the crystal structures. In order to assess the impact of increased template similarity on the accuracy of the homology models we used different sets of templates for modelling, based on their sequence similarity across the entire serpentine domain:

1) Bovine rhodopsin (PDB:1U19) and Japanese flying squid rhodopsin (PDB:2Z73

2) 1U19, 2Z73 and human Adenosine receptor A2A (PDB:3EML)

3) 1U19, 2Z73, 3EML, common turkey Beta-1 adrenergic receptor (PDB;2VT4) and human Beta-2 adrenergic receptor (PDB:2RH1)

In order to assess the impact of using multiple templates for building homology models compared to using the single most similar template, we also built a model for each of the two GPCRs using only the GPCR structure with the highest sequence similarity across the entire serpentine domain, which in both cases was:

4) 2VT4

5) Finally, we used I-TASSER [29], which was ranked 1 in the server category of the Critical Assessment of Structure Prediction (CASP) 2007-2009, to build a model of both drd3_human and cxcr4_human, excluding their respective crystal structures from template selection.

For steps 1 to 4, three models were produced using Modeller 9v7 with the model having the lowest DOPE score selected for further accuracy analysis. I-TASSER produces five models and provides a confidence score (C-score) to indicate the likely best model. Therefore, we chose the I-TASSER model with the highest C-score for further accuracy analysis.

The accuracy of the models generated using the different template sets and methods above were assessed by calculating the RMSD between the C-α residues in the transmembrane region using the align feature of Pymol (Table 1). Unsurprisingly, the results indicate that where multiple templates are used, the addition of more similar templates improves the models e.g. models produced using the two rhodopsin templates (lowest sequence similarity templates to the two targets) had higher RMSDs than the models produced using all five templates. The use of multiple templates compared to a single template produces fairly similar RMSDs, although the multiple template model produced for drd3_human is slightly better than that produced using the single closest template (0.88Å compared to 0.94Å). Furthermore, where the full number of templates are used for modelling with GPCR-SSFE, the model produced for drd3_human is similar in accuracy to that produced by I-TASSER and better than I-TASSER in the case of cxcr4_human.

**Table 1:** The RMSD of cxcr4_human and drd3_human homology models compared to their crystal structures

Method used for template selection	Templates used	Accuracy cxcr4_human (RMSD)^1^	Accuracy drd3_human (RMSD)^2^
Sequence similarity across entire serpentine domain	2VT4	1.72	0.94
Sequence similarity for each TMH	1U19 and 2Z73	1.78	1.21
SSFE workflow or sequence similarity for each TMH	1U19, 2Z73 and 3EML	1.97	1.04
SSFE workflow	1U19, 2Z73, 3EML, 2VT4 and 2RH1	1.73	0.88
I-TASSER	Crystal structure of query protein excluded	1.91	0.82

^1 ^RMSD between cxcr4_human model and 3ODU (TMH region)

^2 ^RMSD between drd3_human model and 3PBL (TMH region)

We also did a comparable analysis of the five template structures and observed similar results to those observed for drd3_human and cxcr4_human, although in some instances the models produced by I-TASSER are significantly worse due to part of the transmembrane domain being modelled on the T4 lysozyme insertion found in some GPCR crystal structures (See additional file 2, Tables S1-S5).

These results validate the effectiveness of the multiple template and fragment approach to homology model building employed by SSFE and indicate that the models are as accurate and, in some cases, better than those produced by one of the current best performing homology modelling programs (as measured by the CASP [30]). We plan to incorporate these two new structures to GPCR-SSFE's workflow and pool of available templates in the very near future and then re-run the template analysis and model building for the 5025 GPCRs in the database.

Our results show that the addition of more similar templates improves the quality of the models and that the single template generated models are similar or slightly worse than the multiple template models. Finally, our results demonstrate that the models produced for the human CXCR4 chemokine receptor and the human dopamine D3 receptor are native like, having RMSDs compared to their crystal structures of 1.7 and 0.9 respectively.

## Discussion

A recent study demonstrated that automated modelling of human neurokinin-1 receptor was enriched by a factor of 2.6 when a combination of bovine rhodopsin and beta-2 adrenergic receptor were used as templates rather than when used singly [31]. Similarly, Mobarec and coworkers found that the use of multiple templates can provide similar or slightly improved models of the transmembrane regions of GPCRs compared to receptor models obtained using single templates [32]. Although a modelling study on opioid receptors demonstrated that it is not always true that a GCPR homology model built using multiple templates is structurally better than those built using one [33].

Our approach to building homology models of GPCRs has a number of advantages over other recent similar studies. Firstly, GPCR-SSFE uses all available family A GPCR templates (that were available at the time of performing the work) to estimate all of the sequence related structural features for a particular receptor and to generate an optimal GPCR model. Secondly, the template for each transmembrane helix is selected individually, meaning that either a single template or a fragment-based approach can be used for model building. This is an important feature considering that it has been shown that sometimes models that are built using one template are better than those built using multiple templates, whereas at other times multiple template models are better [31,32]. Thirdly, GPCR-SSFE has been applied to a very large set of family A GPCRs and all of the homology models have been made available for download and stored in a database. The availability of these initial models allows researchers who are not experienced homology modellers to access three-dimensional data of GPCRs that they are interested in. The user should be aware however, that some GPCR sequences lack particular motifs that are responsible for structural features and which currently occur in all of the available templates, for example, the helix bulges caused by prolines in TMH 5. Since the prolines and bulges are present in all of the templates, they also subsequently occur in the homology models, even when a proline is not present in the corresponding position in a target sequence. Thus, in such cases further refinement of the initial model may be required.

Due to their variability in sequence, length and conformational flexibility, the loop regions of GPCRs are far more difficult to predict by both homology modelling and de novo modelling techniques [34,35]. Based on their dockings studies on human beta-2 adrenergic receptor, Mobarec and coworkers speculate that GPCR models without loops may constitute a better alternative for flexible ligand-rigid protein docking than those with loops [32]. Therefore, although the models built using GPCR-SSFE are loopless, we are confident that they will be extremely useful for i) investigating the mechanisms underlying structural and/or functional effects of mutations; ii) performing docking studies and iii) structure-based drug design. Nevertheless, the provided initial models can be completed by building the loops with suitable software tools [20].

## Conclusions

Our previous analysis indicates that, in general, the structural features of target GPCRs cannot be captured using only one of the experimental GPCR structures as a template for homology modelling. Consequently, we suggest that using transmembrane fragments from multiple templates when building comparative models of GPCRs is likely to lead to more accurate results, an approach that has also been advocated by other published studies on molecular modelling of GPCRs [3,20]. GPCR-SSFE is the first comprehensive GPCR-focused database to store homology models based on the crystallographic advances made in recent years. The fragment-based models stored in GPCR-SSFE serve as a valuable starting-point for molecular analyses of GPCRs based on their structure, such as structure-based drug design or the detailed molecular analysis of mutations. We believe that GPCR-SSFE will be of increasing importance as more crystal structures become available because the problem of template selection will become more complex.

## Availability and requirements

### Availability

GPCR-SSFE is freely available for use on the web at: http://www.ssfa-7tmr.de/ssfe/. The database is publicly and freely accessible, requiring no registration and with no restrictions on use. The GPCR-SSFE server tool requires users to have a Modeller license key, which can be easily and quickly obtained by academic users at the Modeller website http://www.salilab.org/modeller/registration.html.

### Technical requirements

GPCR-SSFE requires that the web browser used supports JavaScript and CSS and that Java is installed and working. It is recommended that one of the following browsers is used: Mozilla Firefox 3 on Linux, Mac OSX or Windows, Internet Explorer 8 on Windows, Safari 4 on Mac OXS or Windows, Chrome on Linux or Windows.

## Abbreviations

CASP: Critical Assessment of Structure Prediction; CSS: cascading style sheets; FAQ: Facts and Questions; GPCR: G protein-coupled receptor; HMM: Hidden Markov Model; HTML: Hypertext Markup Language; LPA: lysophosphatidic acid; MSA: multiple sequence alignment; PDB: Protein Data Bank; TMH: transmembrane helix; RMSD: Root Mean Squared Deviation

## Authors' contributions

CLW performed the algorithm development, homology modelling, designed the database, developed the web interface and wrote the manuscript. AK implemented the search function. CLW and GKr conceived of the study. AK, GKr and GKl conducted the testing. All the authors read and approved the final manuscript.

## Supplementary Material

Additional file 1 **List of the GPCRs in the HMMER profile used for alignment**. List of GPCRs used to build the HMMER profile used for sequence alignment by GPCR-SSFE. The identifier used in the alignment, UniProt entry name, phylogenetic group and cluster (according to the phylogenetic tree for family A [23]) and the receptor type are given. The five GPCRs with crystal structures are highlighted in grey. The amino acid sequences of 3EML, 2VT4 and 2RH1 were modified compared to wild-type (due to either substitutions or deletions) and therefore the wild-type sequences were also included in the profile. Click here for file

Additional file 2 **Accuracy analysis for the five template structures**. Contains the RMSD values for the models produced using the single most similar template, multiple templates of increasing similarity and I-TASSER. Click here for file

## References

[B1] Topiol S Sabio M X-ray structure breakthroughs in the GPCR transmembrane region Biochem Pharmacol 2009 78 11 20 10.1016/j.bcp.2009.02.012 19447219

[B2] Fredriksson R Schioth HB The repertoire of G-protein-coupled receptors in fully sequenced genomes Mol Pharmacol 2005 67 1414 25 10.1124/mol.104.009001 15687224

[B3] Yarnitzky T Levit A Niv MY Homology modeling of G-protein-coupled receptors with X-ray structures on the rise Curr Opin Drug Discov Devel 2010 13 317 25 20443165

[B4] Palczewski K Kumasaka T Hori T Behnke CA Motoshima H Fox BA Trong Le I Teller DC Okada T Stenkamp RE Yamamoto M Miyano M Crystal structure of rhodopsin: A G protein-coupled receptor Science 2000 289 739 45 10.1126/science.289.5480.739 10926528

[B5] Murakami M Kouyama T Crystal structure of squid rhodopsin Nature 2008 453 363 7 10.1038/nature06925 18480818

[B6] Shimamura T Hiraki K Takahashi N Hori T Ago H Masuda K Takio K Ishiguro M Miyano M Crystal structure of squid rhodopsin with intracellularly extended cytoplasmic region J Biol Chem 2008 283 17753 6 10.1074/jbc.C800040200 18463093 PMC2440622

[B7] Warne T Serrano-Vega MJ Baker JG Moukhametzianov R Edwards PC Henderson R Leslie AG Tate CG Schertler GF Structure of a beta1-adrenergic G-protein-coupled receptor Nature 2008 454 486 91 10.1038/nature07101 18594507 PMC2923055

[B8] Cherezov V Rosenbaum DM Hanson MA Rasmussen SG Thian FS Kobilka TS Choi HJ Kuhn P Weis WI Kobilka BK Stevens RC High-resolution crystal structure of an engineered human beta2-adrenergic G protein-coupled receptor Science 2007 318 1258 65 10.1126/science.1150577 17962520 PMC2583103

[B9] Rasmussen SG Choi HJ Rosenbaum DM Kobilka TS Thian FS Edwards PC Burghammer M Ratnala VR Sanishvili R Fischetti RF Schertler GF Weis WI Kobilka BK Crystal structure of the human beta2 adrenergic G-protein-coupled receptor Nature 2007 450 383 7 10.1038/nature06325 17952055

[B10] Jaakola VP Griffith MT Hanson MA Cherezov V Chien EY Lane JR Ijzerman AP Stevens RC The 2.6 angstrom crystal structure of a human A2A adenosine receptor bound to an antagonist Science 2008 322 1211 7 10.1126/science.1164772 18832607 PMC2586971

[B11] Chien EY Liu W Zhao Q Katritch V Han GW Hanson MA Shi L Newman AH Javitch JA Cherezov V Stevens RC Structure of the human dopamine D3 receptor in complex with a D2/D3 selective antagonist Science 2010 330 1091 5 10.1126/science.1197410 21097933 PMC3058422

[B12] Wu B Chien EY Mol CD Fenalti G Liu W Katritch V Abagyan R Brooun A Wells P Bi FC Hamel DJ Kuhn P Handel TM Cherezov V Stevens RC Structures of the CXCR4 chemokine GPCR with small-molecule and cyclic peptide antagonists Science 2010 330 1066 71 10.1126/science.1194396 20929726 PMC3074590

[B13] Worth CL Kleinau G Krause G Comparative sequence and structural analyses of G-protein-coupled receptor crystal structures and implications for molecular models PLoS One 2009 4 e7011 10.1371/journal.pone.0007011 19756152 PMC2738427

[B14] Harmar AJ Hills RA Rosser EM Jones M Buneman OP Dunbar DR Greenhill SD Hale VA Sharman JL Bonner TI Catterall WA Davenport AP Delagrange P Dollery CT Foord SM Gutman GA Laudet V Neubig RR Ohlstein EH Olsen RW Peters J Pin JP Ruffolo RR Searls DB Wright MW Spedding M IUPHAR-DB: the IUPHAR database of G protein-coupled receptors and ion channels Nucleic Acids Res 2009 37 D680 5 10.1093/nar/gkn728 18948278 PMC2686540

[B15] Horn F Weare J Beukers MW Horsch S Bairoch A Chen W Edvardsen O Campagne F Vriend G GPCRDB: an information system for G protein-coupled receptors Nucleic Acids Res 1998 26 275 9 10.1093/nar/26.1.275 9399852 PMC147194

[B16] Satagopam VP Theodoropoulou MC Stampolakis CK Pavlopoulos GA Papandreou NC Bagos PG Schneider R Hamodrakas SJ GPCRs, G-proteins, effectors and their interactions: human-gpDB, a database employing visualization tools and data integration techniques Database (Oxford) 2010 2010 baq019 10.1093/database/baq019 PMC2931634 20689020

[B17] Khelashvili G Dorff K Shan J Camacho-Artacho M Skrabanek L Vroling B Bouvier M Devi LA George SR Javitch JA Lohse MJ Milligan G Neubig RR Palczewski K Parmentier M Pin JP Vriend G Campagne F Filizola M GPCR-OKB: the G Protein Coupled Receptor Oligomer Knowledge Base Bioinformatics 2010 26 1804 5 10.1093/bioinformatics/btq264 20501551 PMC2894509

[B18] Kleinau G Kreuchwig A Worth CL Krause G An interactive web-tool for molecular analyses links naturally occurring mutation data with three-dimensional structures of the rhodopsin-like glycoprotein hormone receptors Hum Mutat 2010 31 E1519 25 10.1002/humu.21265 20513138

[B19] Van Durme J Horn F Costagliola S Vriend G Vassart G GRIS: glycoprotein-hormone receptor information system Mol Endocrinol 2006 20 2247 55 10.1210/me.2006-0020 16543405

[B20] Zhang Y Devries ME Skolnick J Structure modeling of all identified G protein-coupled receptors in the human genome PLoS Comput Biol 2006 2 e13 10.1371/journal.pcbi.0020013 16485037 PMC1364505

[B21] The UniProt Consortium The Universal Protein Resource (UniProt) in 2010 Nucleic Acids Res 2010 38 D142 8 19843607 10.1093/nar/gkp846 PMC2808944

[B22] Durbin R Eddy S Krogh A Mitchison G The theory behind profile HMMs Biological sequence analysis: probabilistic models of proteins and nucleic acids 1998 Cambridge: Cambridge University Press

[B23] Fredriksson R Lagerstrom MC Lundin LG Schioth HB The G-protein-coupled receptors in the human genome form five main families. Phylogenetic analysis, paralogon groups, and fingerprints Mol Pharmacol 2003 63 1256 72 10.1124/mol.63.6.1256 12761335

[B24] Sali A Blundell TL Comparative protein modelling by satisfaction of spatial restraints J Mol Biol 1993 234 779 815 10.1006/jmbi.1993.1626 8254673

[B25] Shen MY Sali A Statistical potential for assessment and prediction of protein structures Protein Sci 2006 15 2507 24 10.1110/ps.062416606 17075131 PMC2242414

[B26] Berman HM Westbrook J Feng Z Gilliland G Bhat TN Weissig H Shindyalov IN Bourne PE The Protein Data Bank Nucleic Acids Res 2000 28 235 42 10.1093/nar/28.1.235 10592235 PMC102472

[B27] Hildebrand PW Goede A Bauer RA Gruening B Ismer J Michalsky E Preissner R SuperLooper--a prediction server for the modeling of loops in globular and membrane proteins Nucleic Acids Res 2009 37 W571 4 10.1093/nar/gkp338 19429894 PMC2703960

[B28] Laskowski RA MacArthur MW Moss DS Thornton JM PROCHECK - a program to check the stereochemical quality of protein structures J. App. Cryst 1993 26 283 91 10.1107/S0021889892009944

[B29] Zhang Y I-TASSER server for protein 3D structure prediction BMC Bioinformatics 2008 9 40 10.1186/1471-2105-9-40 18215316 PMC2245901

[B30] Zhang Y I-TASSER: fully automated protein structure prediction in CASP8 Proteins 2009 77 Suppl 9 100 13 19768687 10.1002/prot.22588 PMC2782770

[B31] Kneissl B Leonhardt B Hildebrandt A Tautermann CS Revisiting automated G-protein coupled receptor modeling: the benefit of additional template structures for a neurokinin-1 receptor model J Med Chem 2009 52 3166 73 10.1021/jm8014487 19397376

[B32] Mobarec JC Sanchez R Filizola M Modern Homology Modeling of G-Protein Coupled Receptors: Which Structural Template to Use? J Med Chem 2009 52 16 5207 16 10.1021/jm9005252 19627087 PMC2891345

[B33] Bera I Laskar A Ghoshal N Exploring the structure of opioid receptors with homology modeling based on single and multiple templates and subsequent docking: A comparative study J Mol Model 2010 Epub ahead of print 10.1007/s00894-010-0803-8 20661609

[B34] Michino M Abola E Brooks CL Dixon JS Moult J Stevens RC Community-wide assessment of GPCR structure modelling and ligand docking: GPCR Dock 2008 Nat Rev Drug Discov 2009 8 455 63 10.1038/nrd2877 19461661 PMC2728591

[B35] Michino M Chen J Stevens RC Brooks CL FoldGPCR: structure prediction protocol for the transmembrane domain of G protein-coupled receptors from class A Proteins 2010 78 2189 201 10.1002/prot.22731 20544957 PMC2933064

