# Supplementary material for: GPCR-SSFE: A comprehensive database of G-protein-coupled receptor template predictions and homology models
Source: BMC Bioinformatics. 2011 May 23;12:185. doi: 10.1186/1471-2105-12-185 (PMC3113946; doi:10.1186/1471-2105-12-185)
Supplement: Additional file 2 — Accuracy analysis for the five template structures. Contains the RMSD values for the models produced using the single most similar template, multiple templates of increasing similarity and I-TASSER. [file 1471-2105-12-185-S2.DOCX]

Table S1. The RMSD of aa2ar_human homology models compared to the crystal structure.

| **Method used for template selection** | **Templates used** | **Accuracy aa2ar_human (RMSD)^1^** |
| --- | --- | --- |
| Sequence similarity across entire serpentine domain | 2VT4 | 1.1 |
| Sequence similarity for each TMH | 1U19 and 2Z73 | 1.7 |
| SSFE workflow or sequence similarity for each TMH | 1U19, 2Z73, 2VT4 and 2RH1 | 1.3 |
| I-Tasser | 3EML excluded | 19.2^*^ |

^1^ RMSD between cxcr4_human model and 3EML (transmembrane helix (TMH) region)

^*^ The second best model according to C-score had an RMSD of 1.1. The poor score for the top model was due to I-Tasser building part of the transmembrane domain using a portion of a GPCR structure that corresponds to a T4 lysozme insertion

Table S2. The RMSD of opsd_bovin homology models compared to the crystal structure.

| **Method used for template selection** | **Templates used** | **Accuracy opsd_bovin (RMSD)^1^** |
| --- | --- | --- |
| Sequence similarity across entire serpentine domain | 2Z73 | 1.4 |
| Sequence similarity for each TMH | 3EML, 2VT4 and 2RH1 | 1.7 |
| SSFE workflow or sequence similarity for each TMH | 3EML, 2VT4, 2RH1 and 2Z73 | 1.5 |
| I-Tasser | All bovine rhodopsin crystal structures excluded | 1.3 |

^1^ RMSD between opsd_bovin model and 1U19 (TMH region)

Table S3. The RMSD of opsd_todpa homology models compared to the crystal structure.

| **Method used for template selection** | **Templates used** | **Accuracy opsd_todpa (RMSD)^1^** |
| --- | --- | --- |
| Sequence similarity across entire serpentine domain | 1U19 | 1.4 |
| Sequence similarity for each TMH | 3EML, 2VT4 and 2RH1 | 1.5 |
| SSFE workflow or sequence similarity for each TMH | 3EML, 2VT4, 2RH1 and 1U19 | 1.4 |
| I-Tasser | All [squid](http://www.pdb.org/pdb/search/smartSubquery.do?smartSearchSubtype=TreeEntityQuery&t=1&n=6637) rhodopsin crystal structures excluded | 4.9^*^ |

^1^ RMSD between opsd_todpa model and 2Z73 (TMH region)

^*^ The second best model according to C-score had an RMSD of 1.4. The poor score for the top model was due to I-Tasser building part of the transmembrane domain using a portion of a GPCR structure that corresponds to a T4 lysozme insertion

Table S4. The RMSD of adrb1_melga homology models compared to the crystal structure.

| **Method used for template selection** | **Templates used** | **Accuracy adrb1_melga (RMSD)^1^** |
| --- | --- | --- |
| Sequence similarity across entire serpentine domain | 2RH1 | 0.5 |
| Sequence similarity for each TMH | 1U19, 2Z73 | 1.7 |
| Sequence similarity for each TMH | 1U19, 2Z73 and 3EML | 1.4 |
| SSFE workflow or sequence similarity for each TMH | 1U19, 2Z73, 3EML and 2RH1 | 0.5 |
| I-Tasser | 2VT4 excluded | 4.4^*^ |

^1^ RMSD between adrb1_melga model and 2VT4 (TMH region)

^*^ The second best model according to C-score had an RMSD of 0.5. The poor score for the top model was due to I-Tasser building part of the transmembrane domain using a portion of a GPCR structure that corresponds to a T4 lysozme insertion

Table S5. The RMSD of adrb2_human homology models compared to the crystal structure.

| **Method used for template selection** | **Templates used** | **Accuracy adrb2_human (RMSD)^1^** |
| --- | --- | --- |
| Sequence similarity across entire serpentine domain | 2VT4 | 0.5 |
| Sequence similarity for each TMH | 1U19, 2Z73 | 1.5 |
| Sequence similarity for each TMH | 1U19, 2Z73 and 3EML | 1.3 |
| SSFE workflow or sequence similarity for each TMH | 1U19, 2Z73, 3EML and 2VT4 | 0.5 |
| I-Tasser | 2RH1 excluded | 0.3 |

^1^ RMSD between adrb2_human model and 2RH1 (TMH region)
